# Psychological distress, intimate partner violence and substance use in a representative sample from Mexico: A structural equation model

**DOI:** 10.3389/fpubh.2023.1101487

**Published:** 2023-03-02

**Authors:** Paola Adanari Ortega Ceballos, Leonor Rivera Rivera, Luz Myriam Reynales Shigematsu, Fernando Austria Corrales, Filiberto Toledano-Toledano, Berenice Pérez Amezcua

**Affiliations:** ^1^Facultad de Enfermería, Universidad Autónoma del Estado de Morelos, Cuernavaca, Morelos, Mexico; ^2^Centro de Investigación en Salud Poblacional, Instituto Nacional de Salud Pública, Cuernavaca, Morelos, Mexico; ^3^Comisión Nacional para la Mejora Continua de la Educación (MEJOREDU), Mexico City, Mexico; ^4^Unidad de Investigación en Medicina Basada en Evidencias, Hospital Infantil de México Federico Gómez, Mexico City, Mexico; ^5^Unidad de Investigación Sociomédica, Instituto Nacional de Rehabilitación Luis Guillermo Ibarra Ibarra, Mexico City, Mexico; ^6^Centro de Investigación Transdisciplinar en Psicología, Universidad Autónoma del Estado de Morelos, Cuernavaca, Morelos, Mexico

**Keywords:** psychological distress, intimate partner violence, alcohol, tobacco, SEM, Mexico

## Abstract

**Introduction:**

Intimate Partner Violence (IPV) is a public health concern associated with multiple adverse health outcomes, including psychological distress (PD).

**Objective:**

To assess the association of IPV and psychological distress, and the mediation of tobacco and alcohol consumption in a national representative sample from Mexico.

**Material and methods:**

Data from the Encuesta Nacional de Consumo de Drogas, Tabaco y Alcohol (ENCODAT) were analyzed. The sample included 34,864 people between the ages of 12 and 65 with a partner. Using Structural Equation Modeling (SEM), the association between IPV, use alcohol, tobacco and psychological distress was measured.

**Results:**

The population was composed of women (51.9%) and men (48.1%); 15.1% (women = 18.2% and men = 11.9%) reported IPV in the last year. The prevalence of psychological distress in the last year was 3.3%, being 3.8% in women, and 2.7% in men. Results from the SEM in women indicated a direct positive effect of the IPV construct on psychological distress (β = 0.298, *p* < 0.01); these findings confirmed that IPV tended to systematically increase psychological distress. Likewise, the presence of IPV increased the consumption of tobacco (β = 0.077, *p* < 0.01) and alcohol (β = 0.072, *p* < 0.01). The SEM results in men showed that alcohol and tobacco consumption tended to increase in the presence of IPV (β = 0.121, *p* < 0.01, and β = 0.086, *p* < 0.01, respectively), and in turn, alcohol consumption and tobacco tended to increase psychological distress (β = 0.024, *p* < 0.01, and β = 0.025, *p* < 0.01, respectively).

**Conclusion:**

This study indicated that in women, IPV had a direct effect on psychological distress and on alcohol and tobacco consumption. Meanwhile in men, alcohol and tobacco consumption had a mediating effect between IPV and psychological distress. The empirical findings of this study will contribute toward the design of public health policies for the prevention and attention of IPV, alcohol and tobacco consumption, and consequently address the mental health consequences derived from these problems.

## 1. Introduction

Psychological distress (PD) refers to maladaptive psychological functioning in the face of stressful life events (1). Characteristics attributed to psychological distress include perceived inability to cope effectively, change in emotional state, discomfort, communication of discomfort, and harm (2).

Various studies conducted in the United States have detected that the prevalence of psychological distress is between 2.9 and 3.9% (3–6). In another study, it was found that the prevalence of psychological distress among African-Americans was 2.1%, in Mexican-Americans 2.0%, and in Latinos 2.6% (7). In Canada, the prevalence of psychological distress was reportedly 8.3%, in women 9.0% and, in men 7.0% (8). A study carried out in Mexico, which used a different version of the psychological distress scale (Kessler 10), found a high prevalence at 67.0% (9).

The relationship between psychological distress and physical and psychological Intimate Violence Partner (IPV) has been documented, mainly in women (6, 10–13). People who reported physical and sexual violence were more likely than those without a history of IPV to report psychological distress. However, when stratified by sex, the risk of psychological distress was higher among women who reported experiencing both physical and sexual IPV. In the United States, 19.9% of women have reportedly experienced IPV compared to 10.9% of men; it has also been reported that women were significantly more likely than men to be classified with PD (3.7 vs. 2.1%) (6).

In a systematic review, 74.0% of the articles investigating the impact of IPV on mental health came from the United States, with the rest of the studies coming from Asia, New Zealand, and Europe; six studies measured the association between the IPV and psychological distress (11). In India, the rate of IPV was reportedly 31.0%. A gradient could be observed between IPV and psychological distress scores; women who reported higher IPV exposure had higher psychological distress scores, while the participants who suffered psychological violence presented an increase of 32.0% in the symptoms of psychological distress (10). Both women and men are at risk of suffering mental health damage associated with IPV; however, these damages can differ according to gender, women present more significant symptoms of depression and post-traumatic stress disorder, and men tend to present anxiety (11).

A study in Spain, carried out with people between the ages of 17 and 23 reported that the coercive behavior of couples weakens the psychological defenses of the victim, with which they can manage to manipulate attitudes and behaviors with the sole purpose of exercising control over the victim (12). In Canada, a study with couples between the ages of 18 and 30 with fewer than five years of relationship, concluded that as women experience more psychological violence, they had higher levels of psychological distress. Regarding physical aggression, it was not significantly correlated with psychological distress; meanwhile men were more likely to report higher levels of psychological distress if they received more psychological or physical violence (13).

IPV is a dysfunctional behavior in which the victim has to adopt coping strategies focused on cognitive, behavioral, or emotional efforts to save themselves from stressors (14, 15). Victims of IPV, in an effort to manage the stressful demands to some coping strategies, turn to coping mechanisms that result in negative health behaviors, such as current smoking and binge drinking (16, 17).

Likewise, it has been found that people with a higher prevalence of current smoking are more likely to have psychological distress compared to non-smokers (5, 16–21). It has also been shown numerous times that binge drinking is significantly associated with increased psychological distress (5, 15, 16, 22, 23). Both current smoking and binge drinking have been associated with psychological distress and therefore could act as mediators between the association of IPV and psychological distress.

People who suffer from IPV (both men and women) can present psychological distress, therefore it is important to identify in a timely manner the main characteristics or factors to define adequate interventions that help people cope positively with IPV and prevent the development of psychological distress.

### 1.1. Current study

The objective of the present study was to estimate the association of IPV and psychological distress, and the mediation of tobacco and alcohol consumption in a representative sample of the Mexican population.

## 2. Material and methods

### 2.1. Sampling and study procedures

**Figure 1:**
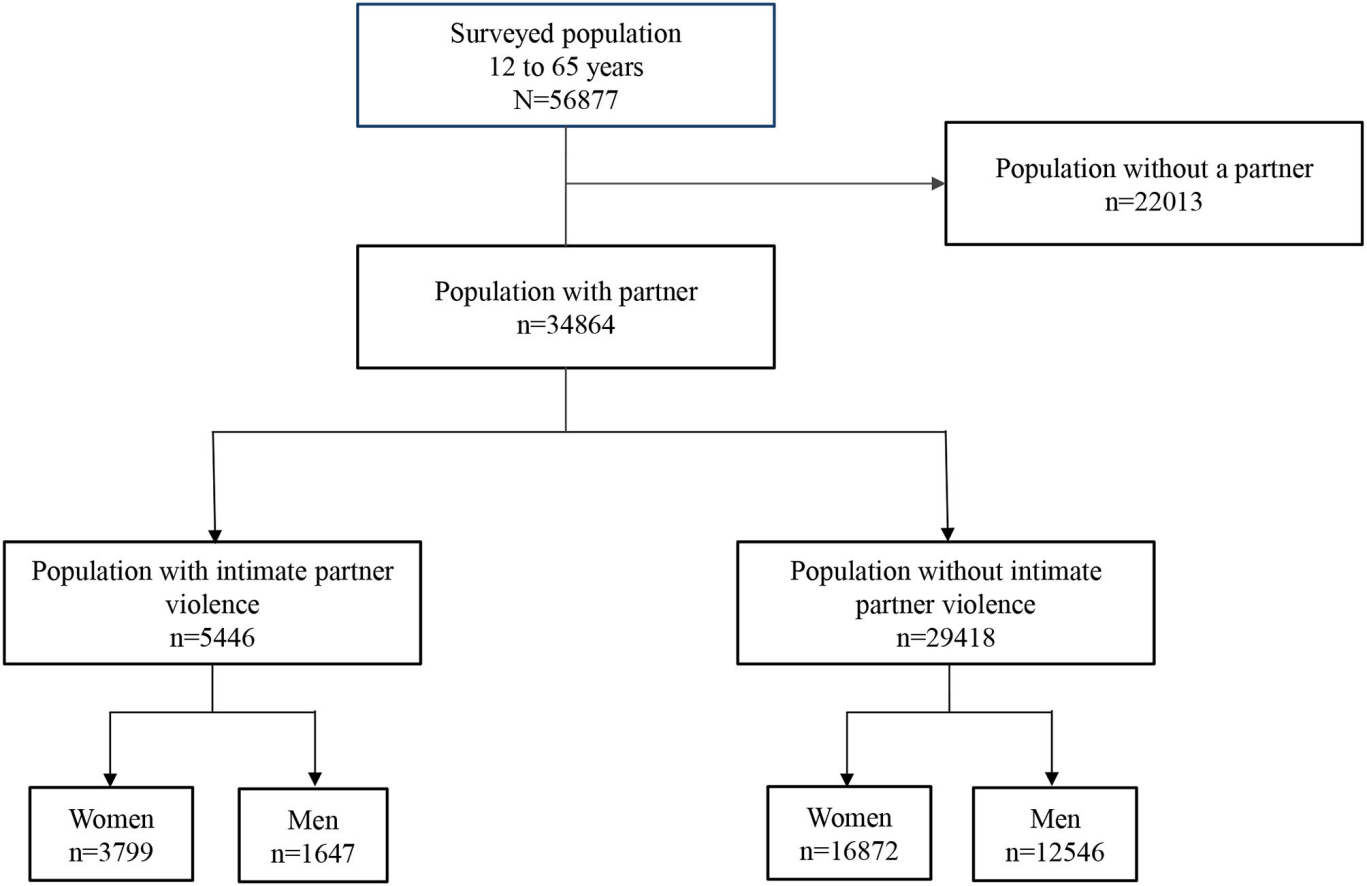
Study population of psychological distress and intimate partner violence. Encuesta Nacional de Consumo de Drogas, Tabaco y Alcohol.

The ENCODAT (24) is a household Survey with a complex design (probabilistic, multi-stage, and stratified sampling by sex, age group, and locality -rural, urban, and metropolitan populations). Households were selected through random sampling; within each household, an adult from 18 to 65 and an adolescent from 12 to 17 were selected. Informed consent was requested from adults, parents, and guardians of minors who participated in the survey. The ENCODAT questionnaire was applied through a face-to-face interview. The sections on IPV and substance use were applied through a computerized self-administered interview strategy (ACASI) (24). The response time for these sections was 20–30 min. The survey had a standardized methodology; the interviewers had experience applying national health surveys and were trained and supervised throughout the fieldwork. The global response rate was 73.64% (24). For the present study, the sample included a population with a history of a partner (*n* = 34,864). Of these, 3,799 were women, and 1,647 were men (Figure 1). The study was approved by the ethics committees of the National Institute of Psychiatry RFM and the National Institute of Public Health (Conbioética: 17CEI00120130424; Cofepris:13 CEI 17 007 36; FWA: 00015605) (24).

### 2.2. Measures

#### 2.2.1. Psychological distress

The Kessler Psychological Distress Scale-6 (K6), was used to measure the extent and the severity of generalized distress in the preceding month (25). This scale has been validated in the Mexican population (Cronbach's alpha = 0.83) (26). The scale contains six items: (a) how often did you feel nervous (Nrv)?, (b) how often did you feel hopeless (Hop)?, (c) how often did you feel restless or fidgety (Rst)?, (d) how often did you feel so sad that nothing could cheer you up (Chr)?, (e) how often did you feel that everything was an effort (Eff)?, and (f) how often did you feel worthless (Ngd)? (27). Each item of the K6 is ranged on a 5-point Likert-type intensity scale: None of the time; A little of the time; Some of the time; Most of the time, and All of the time. The items of the K6 are scored from 0 to 4 and the total score is the sum of these responses which ranges from 0 to 24. Respondents were classified as having severe psychological distress if they scored 13 or greater. For the purposes of this study, the scale was validated and Cronbach's alpha = 0.87 was obtained.

#### 2.2.2. Intimate partner violence scale

The variable of partner violence was constructed during the last 12 months, considering eight items: (a) has your partner shouted at you, insulted you, or humiliated you (Sh); (b) has threatened to beat you (Thb); (c) has beaten you (Bt); (d) has forced you to have sexual intercourse (Sx); (e) controls or has controlled most of your activities (Cn); (f) manifests his/her jealousy by beating (Jl), (g) has threatened to commit suicide or has attempted it (Sc); (h) “has controlled you by not giving you money for household expenses or by taking it away from you?” (Mn) (28). Each item of IPV had a dichotomous response (no = 0; yes = 1). Subsequently, the items were added, obtaining a discrete variable, the total score of the sum of these responses ranged from 0 to 7. Respondents were classified as having Intimate Partner Violence if they scored 1 or greater, and finally, there was a dichotomous variable for IPV (0 = without violence and 1 = with violence). This scale has been previously validated by Natera et al. (28) (Alfa de Cronbach= 0.76).

#### 2.2.3. Binge drinking

This variable was defined as alcohol consumption of five drinks or more on a single occasion for men and four drinks or more on a single occasion for women in the last month (29).

#### 2.2.4. Current smoking in the last year

The variable of tobacco consumption was constructed with the following questions: when was the last time you smoked a cigarette? Do you currently smoke tobacco every day, some days, or do you currently not smoke? People who had smoked tobacco in the last year were categorized as smokers and the population that had not smoked tobacco in the last year as non-smokers (30).

#### 2.2.5. Age

Categorized in 14–17, 18–28, 29–39, 40–59, and 60 and over years of age.

#### 2.2.6. Education

Categorized into no formal education, primary, secondary, high school, and college.

#### 2.2.7. Socioeconomic status

It was estimated by constructing an index of household assets, in which the first quintile corresponds to the lowest socioeconomic level and the fifth to the highest (24).

### 2.3. Statistical analysis

The interest of the study was to analyze the mediating role of alcohol and tobacco consumption in the association between IPV and PD (31); therefore, an approach based on structural equation modeling was used. Other alternatives, such as multivariate regression, do not allow the modeling of the covariance structure matrix with the necessary flexibility to estimate the direct and indirect effects of the variables involved in the proposed theoretical model, given that one of the basic assumptions of multivariate regression is the absence of collinearity between the independent variables. In the present study, such assumption would not be fulfilled. Additionally, structural equation modeling allows modeling variances and covariances of latent variables or factors. In the present study, these are present in the variables of IPV and psychological distress, which further explain the covariance matrix of other manifest variables in the model.

Considering the above, the covariance matrix between IPV, PD, Bd, Cs, Age, Edu, and SES was analyzed using the maximum likelihood method. Four fit indices were used to assess the model: Comparative Fit Index (CFI) takes possible values between 0 and 1, considering a value of at least 0.90 denotes an adequate fit of the model, while a value ≥0.95 shows a very good fit. The Tucker – Lewis Index (TLI) and the Bentler-Bonnet Normed Fit Index (NFI) both with a range between 0 and 1 with interpretation values like the CFI, the Root Mean Square Error Approximation (RMSEA) should ideally have values less than 0.06, however, values of 0.08 are also considered acceptable (32, 33).

Initially, the IPV and psychological distress constructs were adjusted using CFA, then two structural equation models were built, one for women and the other for men, associating IPV with psychological distress and using Bd and Cs as mediating variables and Age, Edu, and SES as covariates. Finally, the two models were compared.

## 3. Results

### 3.1. Sample's characteristics

In the sample, 51.9% were women and 48.1% men. Of the participants, 35.6% were between 40 and 59 years old, and 9.9% with university studies. More than 20.0% of the population was found in the highest quintile of socioeconomic status. The prevalence of psychological distress in the last year was 3.3%, being 3.8% in women and 2.7% in men, showing a significant difference by sex (*p* < 0.05, Chi-squared test). Of the population, 15.1% presented IPV in the last year (women = 18.2% and men = 11.9%, *p* < 0.001). The percentage of the population that reported tobacco consumption in the last year was 22.3%, presenting a higher prevalence in men with 34.4% (*p* < 0.001, Chi-squared test). Regarding alcohol consumption, 9.8% mentioned excessive consumption in the last year, with a consumption in men of 16.5% (*p* < 0.001, Chi-squared test) (Table 1).

**Table 1:** Study population characteristics.

	**Women** ***n*** = **20,671**		**Men** ***n*** = **14,193**		**Chi^2^**
**Characteristics**	**%**	**CI 95%^b^**	**%**	**CI 95%**	***p***
**Psychological distress**					
No	96.2	(95.7–96.6)	97.3	(96.3–98)	0.044
Yes	3.8	(3.4–4.3)	2.7	(2–3.7)	
**Age (years)**					
14–17	5.5	(5–6)	5.4	(5–5.9)	< 0.001
18–28	27.8	(26.6–29.1)	24.6	(23.2–25.9)	
29–39	26.9	(25.9–27.9)	24.0	(22.9–25.2)	
40–59	34.1	(33–35.3)	37.1	(35.7–38.5)	
60 y más	5.7	(5.2–6.2)	8.9	(7.9–10)	
**Education**					
No formal education	2.7	(2.3–3.1)	2.5	(2.1–3)	< 0.001
Primary	30.5	(29.3–31.7)	29.6	(28.3–31.1)	
Secondary	36.9	(35.8–38)	33.4	(31.9–34.9)	
High school	21.4	(20.3–22.6)	23.1	(21.8–24.5)	
College or more	8.5	(7.8–9.3)	11.4	(10.4–12.5)	
**Socioeconomic status**					
First	16.7	(15.8–17.6)	16.5	(15.5–17.6)	< 0.001
Second	21.5	(20.6–22.5)	19.6	(18.5–20.7)	
Third	20.1	(19.2–21.1)	18.4	(17.2–19.6)	
Fourth	21.0	(19.9–22.1)	20.7	(19.5–21.8)	
Fifth	20.8	(19.5–22.1)	24.9	(23.3–26.6)	
**Intimate partner violence**					
No	81.8	(80.9–82.8)	88.1	(87.2–89)	< 0.001
Yes	18.2	(17.2–19.1)	11.9	(11–12.8)	
**Current smoking in the last year**					
No	88.9	(88–89.6)	65.6	(64.2–67)	< 0.001
Yes	11.2	(10.4–12)	34.4	(33–35.8)	
**Binge drinking**					
No	96.3	(95.8–96.8)	83.5	(82.3–84.7)	< 0.001
Yes	3.7	(3.2–4.2)	16.5	(15.3–17.7)	

ENCODAT^a^ México.

^a^Encuesta Nacional de Consumo de Drogas, Tabaco y Alcohol (ENCODAT).

^b^CI 95%, Confidence interval.

Those who reported psychological distress in the last year, 60.4% were women, 38.4% belonged to the age group of 40 to 59 years (women = 40.7% vs. men = 35.0%), 26.4% were located in the second quintile of SES, women with 27.1% and men with 25.2%, 45.0% reported IPV in the last year (women = 50.9% and men = 33.8%, *p* < 0.05), 30.7% used tobacco (women = 19.0% and men = 48.4%, *p* < 0.001) and 18.6% mentioned binge drinking (women = 8.2% and men = 34.4%, *p* < 0.001; Table 2).

**Table 2:** Study population psychological distress.

	**Women** ***n*** = **20,671**		**Men** ***n*** = **14,193**		**Chi^2^**
**Characteristics**	**%**	**IC 95%^b^**	**%**	**IC 95%**	***p***
**Age (years)**					
14–17	10.2	(6.5–15.7)	7.4	(4.2–12.7)	0.1995
18–28	21.1	(16.6–26.4)	26.3	(15.5–40.8)	
29–39	21.4	(16.3–27.5)	13.8	(8.2–22.3)	
40–59	40.7	(35.4–46.2)	35.0	(23.4–48.7)	
60 years and over	6.7	(4.3–10.2)	17.5	(4.4–49.7)	
**Education**					
No forma3.1l education	4.0	(2.5–6.4)	3.9	(1.8–8.1)	0.6645
Primary	44.2	(38.7–49.8)	49.8	(33.9–65.7)	
Secondary	32.4	(27.6–37.7)	32.2	(20.5–46.6)	
High school	15.6	(10.6–22.4)	12.6	(7.2–21.2)	
College or more	3.7	(2–6.9)	1.5	(0.7–3.3)	
**Socioeconomic status**					
First	21.4	(17.7–25.7)	20.5	(13.4–30.1)	0.3922
Second	27.1	(21.4–33.7)	25.2	(14.9–39.4)	
Third	18.7	(14.8–23.4)	29.9	(14.1–52.5)	
Fourth	20.9	(16.3–26.5)	12.8	(7.2–21.6)	
Fifth	11.8	(8.3–16.5)	11.7	(6.1–21.3)	
**Intimate partner violence**					
No	48.4	(42.4–54.3)	65.2	(51.4–76.9)	0.0294
Yes	51.6	(45.7–57.6)	34.8	(23.1–48.7)	
**Current smoking in the last year**					
No	81.0	(73.8–86.6)	51.6	(36.1–66.8)	< 0.001
Yes	19.0	(13.4–26.2)	48.4	(33.3–63.9)	
**Binge drinking**					
No	91.8	(86–95.4)	65.6	(44.9–81.7)	< 0.001
Yes	8.2	(4.6–14)	34.4	(18.3–55.2)	

ENCODAT^a^ México.

^a^Encuesta Nacional de Consumo de Drogas, Tabaco y Alcohol (ENCODAT).

^b^CI 95% -Confidence interval.

#### 3.1.1. Structural equation model between IPV and PD mediated by Bd and Cs: Women

The results showed an absolute fit of *X*^2^ = 4137.89, *p* < 0.001 and the following fit indices: CFI = 0.961, TLI = 0.953, NFI = 0.960, and RMSEA = 0.03 (0.036–0.038), such that the model was considered to have a good fit and there were no significant differences between the theoretical model and the empirical data. The standardized parameters obtained in the model are shown in Table 3.

**Table 3:** Structural equation model between IPV and PD mediated by Bd and Cs: standardized parameters in women.

**Factor loadings**	**Estimation**	**Wald Z**	**Prob > |Z|**
IPV → Sh	1	.	.
IPV → Thb	0.671	78.137895	< 0.0001
IPV → Bt	0.553	64.319071	< 0.0001
IPV → Sx	0.434	52.214899	< 0.0001
IPV → Cn	0.622	70.775402	< 0.0001
IPV → Jl	0.702	76.533969	< 0.0001
IPV → Sc	0.343	42.463643	< 0.0001
IPV → Mn	0.568	66.448215	< 0.0001
PD → Nrv	1	.	.
PD → Hop	0.728	78.553436	< 0.0001
PD → Rst	0.691	92.916392	< 0.0001
PD → Chr	0.809	81.12735	< 0.0001
PD → Eff	0.785	79.829017	< 0.0001
PD → Ngd	0.709	74.67298	< 0.0001
**Regressions**	**Estimation**	**Wald Z**	**Prob** > **|Z|**
IPV → PD	0.298	33.184217	< 0.0001
IPV → Bd	0.072	9.2616639	< 0.0001
IPV → Cs	0.077	9.8763654	< 0.0001
Bd → PD	0.014	1.9204759	0.0548
Cs → PD	0.052	6.994088	< 0.0001
Age → IPV	0.009	1.1773236	0.2391
Age → PD	0.016	2.2114943	0.0270
Edu → IPV	0.016	1.8202702	0.0687
Edu → PD	0.128	16.021357	< 0.0001
SES → IPV	0.067	7.9967447	< 0.0001
SES → PD	0.023	2.9872334	0.0028

SES, Socioeconomic status; Edu, Education; PD, Psychological distress; Bd, Binge drinking; Cs, Current smoking; IPV, Intimate partner violence; Sh, Shout; Thb, threatened to beat; Bt, Beat to you; Sx, Force sex intercourse; Ji, Jealousy; Sc, Suicidal ideation; Mn, Money control.

In the resulting model it was possible to appreciate the direct positive effect that the IPV construct has on psychological distress (β = 0.298, *p* < 0.01), which suggests that IPV systematically tends to increase psychological distress in women. Likewise, it was observed that the presence of IPV tended to increase the consumption of tobacco (β = 0.077, *p* < 0.01) and alcohol (β = 0.072, *p* < 0.01); these effects, although of small magnitude, they were statistically significant. In the case of tobacco consumption, it was possible to confirm that there was a mediating effect between IPV and psychological distress (β = 0.052, *p* < 0.001), while alcohol consumption had no significant effect on psychological distress (β = 0.014, *p* < 0.0548).

#### 3.1.2. Structural equation model between IPV and psychological distress mediated by Bd and Cs: Men

The theoretical model and corresponding estimates that were hypothesized for women were also used for men. The absolute fit of *X*^2^ = 2350.07, *p* < 0.001 and the following fit indices, CFI = 0.963, TLI = 0.955, NFI = 0.961 and an RMSEA = 0.03 (0.032–0.034), therefore it was considered a model with good fit, and it was possible to infer that there were no significant differences between the theoretical model and the data. The standardized parameters are shown in Table 4.

**Table 4:** Structural equation model between IPV and PD mediated by Bd and Cs: standardized parameters in men.

**Factor loadings**	**Estimation**	**Wald Z**	**Prob > |Z|**
IPV → Sh	1	.	.
IPV → Thb	0.615	51.873574	< 0.0001
IPV → Bt	0.545	45.528168	< 0.0001
IPV → Sx	0.315	29.76013	< 0.0001
IPV → Cn	0.541	45.940913	< 0.0001
IPV → Jl	0.626	49.851903	< 0.0001
IPV → Sc	0.332	31.140388	< 0.0001
IPV → Mn	0.397	36.602369	< 0.0001
PD → Nrv	1	.	.
PD → Hop	0.736	65.135725	< 0.0001
PD → Rst	0.665	75.417528	< 0.0001
PD → Chr	0.808	66.523551	< 0.0001
PD → Eff	0.769	64.857219	< 0.0001
PD → Ngd	0.707	61.198114	< 0.0001
**Regressions**	**Estimation**	**Wald Z**	**Prob** > **|Z|**
IPV → PD	0.228	20.461203	< 0.0001
IPV → Bd	0.121	12.253285	< 0.0001
IPV → Cs	0.086	8.7365848	< 0.0001
Bd → PD	0.024	2.6089394	0.0091
Cs → PD	0.025	2.750778	0.0059
Age → IPV	−0.021	−2.116186	0.0343
Age → PD	−0.04	−4.48178	< 0.0001
Edu → IPV	−0.027	−2.478551	0.0132
Edu → PD	0.131	13.217565	< 0.0001
SES → IPV	0.041	3.8164241	0.0001
SES → PD	0.03	3.1179137	0.0018

SES, Socioeconomic status; Edu, Education; PD, Psychological distress; Bd, Binge drinking; Cs, Current smoking; IPV, Intimate partner violence; Sh, Shout; Thb, threatened to beat; Bt, Beat to you; Sx, Force sex intercourse; Ji, Jealousy; Sc, Suicidal ideation; Mn, Money control.

These results were similar to the findings obtained in the women group; however, the magnitude of the regression between IPV and psychological distress was smaller in contrast to the parameter estimated in women (β = 0.228, *p* < 0.01 in men vs. β = 0.298, *p* < 0.01 in women), suggesting that, in women, IPV systematically increases the risk of psychological distress more than in men. Likewise, it is possible to observe that, in the group of men, tobacco and alcohol consumption mediate a small portion of the covariance between IPV and psychological distress.

In the present study, alcohol consumption tended to increase in the presence of IPV (β = 0.121, *p* < 0.01), as well as tobacco consumption (β = 0.086, *p* < 0.01) and, in turn, alcohol consumption tended to increase psychological distress (β = 0.024, *p* < 0.01) similarly to the case of tobacco (β = 0.025, *p* < 0.01). The effects identified in both models were controlled by age, SES, and education, suggesting that these direct and indirect effects could be accurate in the construction of predictive models.

## 4. Discussion

This study, based on data from a representative sample of Mexico, confirmed a relationship between IPV and psychological distress. Additionally, it was confirmed that the consumption of tobacco and/or alcohol has a mediating effect between IPV and psychological distress, mainly in men.

The results of the present study demonstrated the direct effect of IPV on psychological distress, these data are consistent with Lagdon et al. where they found that women with IPV were more likely to report higher psychological distress compared to those who did not experience violence (11). This effect has been studied in different populations, in which they confirm the direct relationship between these two variables (6, 10, 12, 13). The increasing adverse effects of IPV on the mental health of victims, compared to those who have never experienced IPV have been widely documented. IPV has been associated with anxiety, depression, substance abuse (34), and post-traumatic stress disorder (34, 35). It has been shown that IPV is positively associated with the severity of posttraumatic stress disorder symptoms, finding a greater association in women who reported having been victims of psychological violence. A plausible explanation for these findings could be that the experience of psychological IPV can provoke unique response patterns that exacerbate emotional dysregulation, generating psychological distress (36). Although the damage caused by IPV can occur in anyone with IPV, it has been documented that the damage is greater in women than in men.

According to the results of the present study, it was confirmed that IPV has a direct and mediating effect by tobacco and/or alcohol consumption toward psychological distress, although the mediating effects were not of great magnitude, they were significant. The consumption of alcohol or tobacco tends to increase the psychological distress; these results are consistent with the literature, which reports that people with IPV can develop coping skills (37), such as tobacco and alcohol consumption (17); however, these behaviors have been associated with psychological distress (16).

The consumption of tobacco and alcohol, as a consequence of IPV, reflects the importance of considering sex as a category of analysis (38). Women are socially allowed to express their emotions more, while in men, there is no recognition of these emotions, leading to the consumption of alcohol and tobacco (39). The results obtained are similar to those in other studies, which report that people who experience psychological distress are more likely to have a higher prevalence of smoking than the general population (5, 18–21).

Another study Sung et al. conducted in the United States population found an association between tobacco use and psychological distress. They concluded that people with psychological disorders were more likely to be current smokers and tended to be heavy smokers once they started smoking (5). Thus, they detected a significant increase in psychological distress among current smokers; they also observed a higher prevalence of psychological distress among younger smokers with less formal education and lower annual family income. It has been documented that reduced rates of psychological distress among ex-smokers may suggest that smoking played a role in the maintenance of psychological distress and the increased likelihood of remission after a successful quit attempt (19). Hagman et al. found that adults with psychological distress were more likely to use tobacco in their lifetime than those without psychological distress (21). Likewise, in the Australian population, it was observed that current smokers, especially those who smoked daily, presented higher levels of psychological distress (20). In the Japanese population (40), when carrying out the analysis stratified by sex, a significant positive association was found in women between tobacco use and psychological distress but not in men.

Our results show that the effect of IPV on alcohol consumption tends to be greater in men than in women, Nakagawa et al. (41) reported results that coincide with ours, in which men who consume alcohol presented a higher risk of psychological distress compared to women. However, these results differ from what was reported by Øverup, in which the coefficient indicated that the effect was stronger for women than for men (42). Various studies have reported a positive association between binge drinking and psychological distress. A study in the United States (23), reported a significant association between binge drinking and higher levels of psychological distress. In Japan, Nakagawa et al. (41) the same trend was observed, the greater the consumption of alcohol, the greater the presence of psychological distress, which coincides with the empirical findings in the present study.

Although this study makes valuable contributions to understand the role of IPV in psychological distress among women and men experiencing IPV, the findings must be interpreted in the context of certain limitations. First, the cross-sectional nature of the data includes determining the nature and not attributing causality in the direction of the relationships examined. Further studies are needed to investigate these relationships through prospective and longitudinal investigations. A second limitation is the use of a secondary database, in which the instrument did not measure the severity of IPV; although we assume that all violent practices have health consequences. Third, this study relied on people's self-report of psychological distress symptoms, which may have been influenced by their ability or willingness to report accurately.

Another limitation is that the ENCODAT did not include information about abuse in childhood. Previous research suggests childhood abuse is associated with an increased risk of psychological distress as it has been observed to affect gender attitudes and power in sexual relations since it is a crucial determinant of adverse outcomes in adult life. This is consistent with the literature showing that men who were abused as children are more likely to have witnessed parental violence and have been socialized into unequal gender norms that lead to the use of violence in their intimate relationships. Therefore, it is vital to account for childhood abuse as a crucial area of action to prevent mental health issues, the development of negative attitudes, and the intergenerational transmission of violence (43).

Machisa et al. (44), with a South African population, observed that child abuse had direct effects on post-traumatic stress disorder symptoms and depression, as well as indirect effects on excessive alcohol consumption, showing that exposure to violence during childhood can have adverse effects. In mental health, it promotes the intergenerational transmission of this behavior to adult life (44). It would also be essential to have information on victims and perpetrators, both female and male.

Future research should be longitudinal, through cohort studies, the temporality between the appearance of psychological distress and exposure to various variables could be observed, being certain that the exposure preceded the event.

A notable strength of the study is the representativeness of the Mexican population. The process of data collection has advantages for the generalizability of results, due to the control in the research setting for various demographic characteristics. Moreover, these results are consistent with other investigations; therefore, it can be inferred that they have internal and external validity.

The results from this study provide evidence to strengthen the existing information on the relevance of directing preventive strategies that promote the mental health of women and men who have been victims of IPV. It also underscores the importance of using sex as a category of analysis with the purpose of reducing the consumption of alcohol and tobacco, providing adequate tools to avoid risks that threaten health outcomes.

It is essential that IPV be dismantled through actions that transform the context of inequality between women and men (45) through the creation of spaces free of violence that promote equity, justice, and good treatment. This is vital to avoid negative consequences on mental health and the excessive consumption of alcohol and tobacco in the population.

The pattern of IPV reported in this study reveals a worrying panorama that requires a multidisciplinary approach considering social, economic, and intercultural differences in Mexico. A potential move forward would be the implementation of psychological distress screenings within healthcare centers, workplaces, and schools to identify the population at risk and to provide timely care.

## 5. Conclusion

The findings of this study have important public health implications. It provides helpful information to further identify and understand the effects of IPV on women's and men's mental health. These findings further emphasize the need for programing and public health policies for IPV prevention.

Knowledge about the effects of excessive alcohol consumption and tobacco use, and their relationship with psychological distress, provides information to healthcare personnel to identify people who are at risk. In turn, this allows them to develop comprehensive care interventions (i.e., mental health and substance use prevention). More broadly, this information serves to sensitize the general population regarding the need to prioritize and position these problem in the public agenda.

## Data availability statement

The raw data supporting the conclusions of this article will be made available by the authors, without undue reservation.

## Ethics statement

The studies involving human participants were reviewed and approved by Conbioética: 17CEI00120130424; Cofepris:13 CEI 17 007 36. The patients/participants provided their written informed consent to participate in this study.

## Author contributions

LRe, LRi, and PO: conceptualization and methodology. LRe, LRi, FA, and PO: software. FA: validation and formal analysis. LRe, LRi, PO, and FT-T: investigation. FA and PO: data curation. LRe, LRi, BP, and PO: writing—original draft preparation. LRe, PO, and FT-T: writing—review and editing. All authors have read and agreed to the published version of the manuscript.
